# Unveiling the Neurotoxicity of Metronidazole: A Clinical Conundrum

**DOI:** 10.7759/cureus.62219

**Published:** 2024-06-12

**Authors:** Gollapudi Hithesh, Swathy Moorthy, Lakshmi M, Emmanuel Bhaskar

**Affiliations:** 1 General Medicine, Sri Ramachandra Institute of Higher Education and Research, Chennai, IND; 2 Internal Medicine, Sri Ramachandra Medical College, Sri Ramachandra Institute of Higher Education and Research, Chennai, IND; 3 Internal Medicine, Sri Ramachandra Institute of Higher Education and Research, Chennai, IND

**Keywords:** carcinomatous neuropathy, dentate nucleus, peripheral neuropathy, hepatic abscess, metronidazole

## Abstract

Metronidazole, a commonly used antiprotozoal agent, has been linked to neurotoxicity in a few individuals. We present the case of a 61-year-old gentleman diagnosed with a liver abscess, who received a total dose of 64 g of metronidazole over a four-week duration. He subsequently developed slurred speech, numbness, and tingling sensation in both feet. His neuroimaging revealed T2 hyperintensities in the bilateral dentate nuclei and withdrawal of the drug led to symptomatic improvement in the patient. Metronidazole is known to produce neurological manifestations with involvement of peripheral nerves and cerebellum commonly. In the present case, the cumulative dose impact of metronidazole on the dentate nucleus was evident.

## Introduction

Metronidazole (1-(β-hydroxy-ethyl)-2-methyl- 5-nitroimidazole) is a commonly used antibiotic for anaerobic and protozoan infections. The most common side effects of metronidazole are nausea, vomiting, diarrhea, and a slight metallic taste in the mouth. If an individual consumes alcohol while on the drug, it can precipitate side effects such as nausea, vomiting, abdominal bloating, hot flashes, shortness of breath, palpitations, and headaches. However, the occurrence of neurologic symptoms, particularly peripheral neuropathy predominantly involving the lower limbs, with prolonged therapy has been uncommonly reported [1,2]. Treatment with metronidazole for more than four weeks and with a cumulative dose greater than 42 g poses a risk of developing peripheral neuropathy [2]. Here, we present a recently identified case of peripheral neuropathy due to prolonged metronidazole administered for the treatment of liver abscess.

## Case presentation

**Figure 1:**
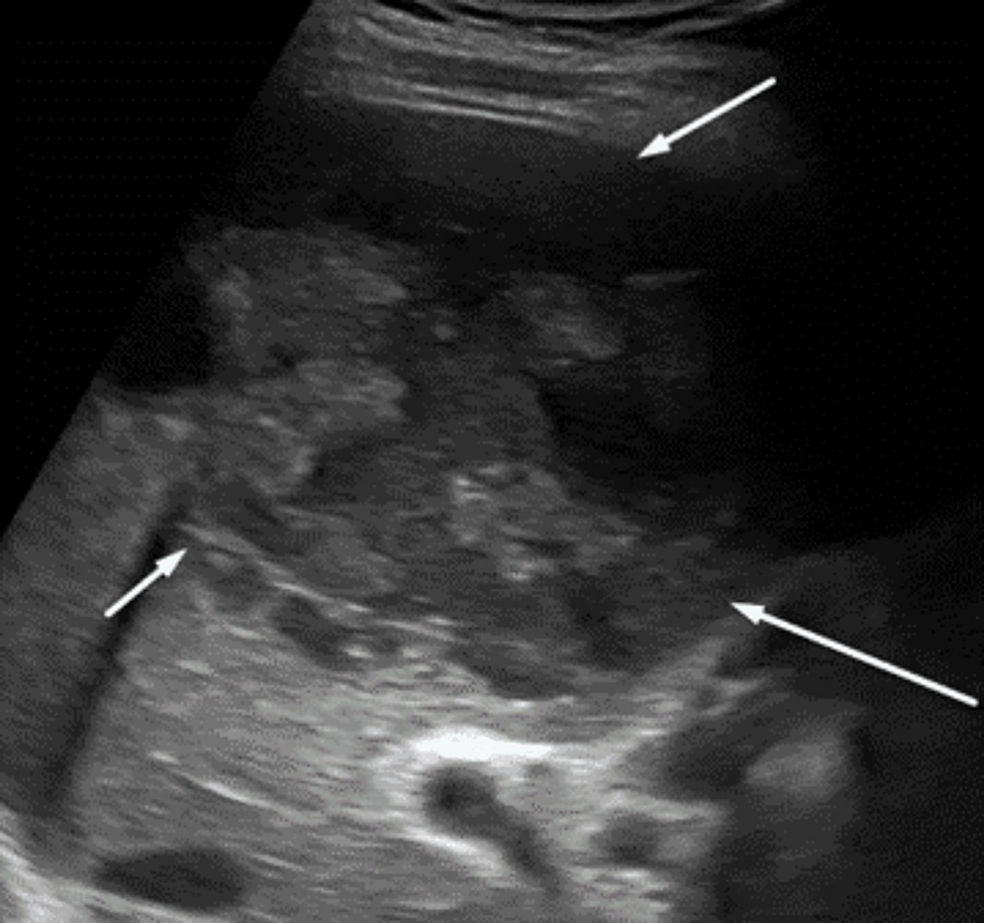
Ultrasound of the abdomen showing liver abscess at admission.

A 61-year-old gentleman with diabetes and coronary artery disease presented to our outpatient department with an eight-day history of high-grade fever with chills. Examination revealed right hypochondriac tenderness and other systemic examinations were unremarkable. His investigations revealed elevated total counts with polymorph predominance (14,470 cells/mm^3^; 80% polymorphs). Liver and kidney function tests were normal except for mild transaminitis (serum glutamic-oxaloacetic transaminase: 58 U/L and serum glutamic-pyruvic transaminase: 76 U/L). Ultrasonography revealed two ill-defined heterogeneous lesions with solid cystic components in both lobes of the liver with increased vascularity and 109 mL and 84 mL volume, respectively (Figure 1).

**Figure 2:**
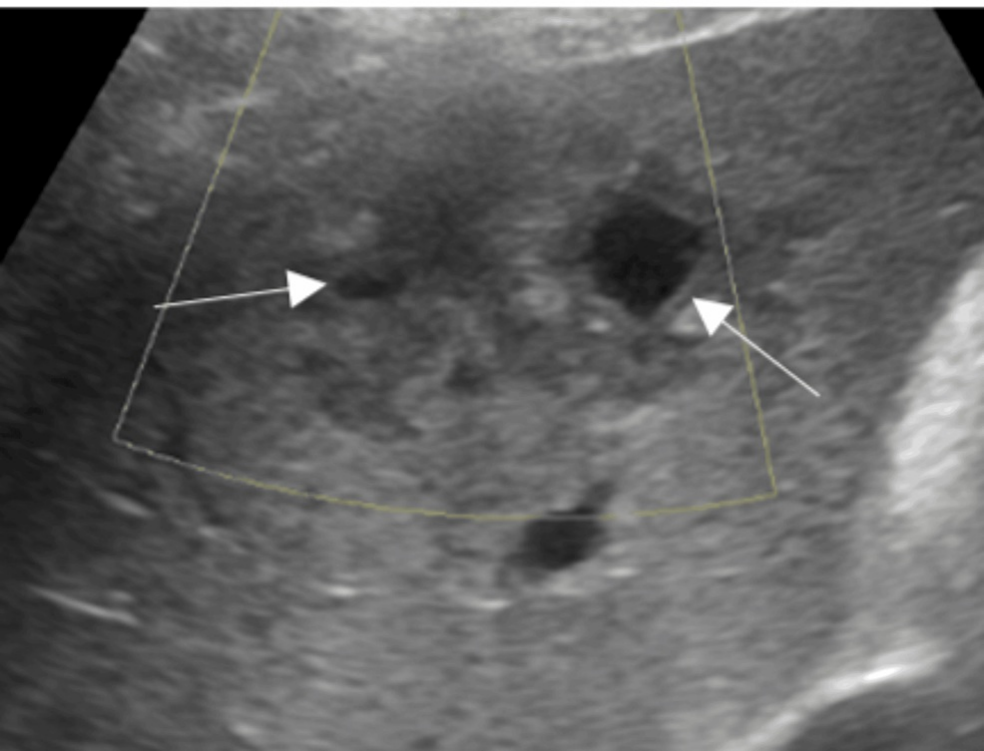
Ultrasound of the abdomen showing liver abscess displaying a regression in size.

Because of the surrounding vascularity and an incomplete liquefaction, ultrasound-guided pigtail insertion and drainage of the collection could not be performed. The patient was managed conservatively with tablet metronidazole 800 mg thrice a day. He received a cumulative dose of 34 g over two weeks in the hospital and 34 g for two weeks post-discharge. At discharge, screening ultrasound revealed a decrease in abscess size to 74 mL and 72 mL, respectively (Figure 2).

During the review, the patient complained of numbness in both soles, which gradually progressed to above the ankles over seven days. He also gave a history of falling from his bike due to difficulty operating the gear with his foot. Examination revealed loss of touch and vibration sensations in both lower limbs, with no other remarkable finding. Nerve conduction studies (NCS) revealed motor axonal neuropathy of the upper limbs with bilateral median nerve entrapment neuropathy at the wrist, and motor radiculo-neuropathy in both lower limbs. The results of the NCS are shown in Tables 1, 2.

**Table 1:** Nerve conduction study results of both upper limbs. NCV = nerve conduction velocity

	Latency (ms)	Amplitude (mV)	NCV (m/s)
Right median	4.38	4.2	51.28
Left median	4.27	4.5	51.20
Right ulnar	3.02	9.5	68
Left ulnar	2.92	8.0	66.67

**Table 2:** Nerve conduction study results of both lower limbs. NCV = nerve conduction velocity

	Latency (ms)	Amplitude (mV)	NCV (m/s)
Right peroneal	4.69	6.5	45.14
Left peroneal	4.06	4.1	44.02
Right tibial	4.48	16.2	44.71
Left tibial	5.21	14.0	45.25

**Figure 3:**
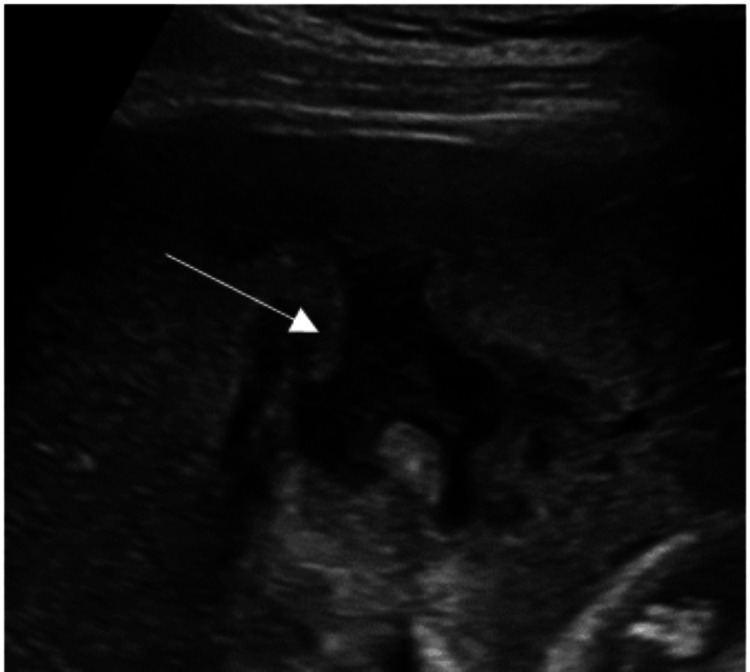
Follow-up ultrasound showing an increase in the size of the abscess.

The follow-up abdominal ultrasound revealed that the abscess had almost doubled in size to 130 mL (Figure 3) while on treatment, leading to a suspicion of malignancy.

**Figure 4:**
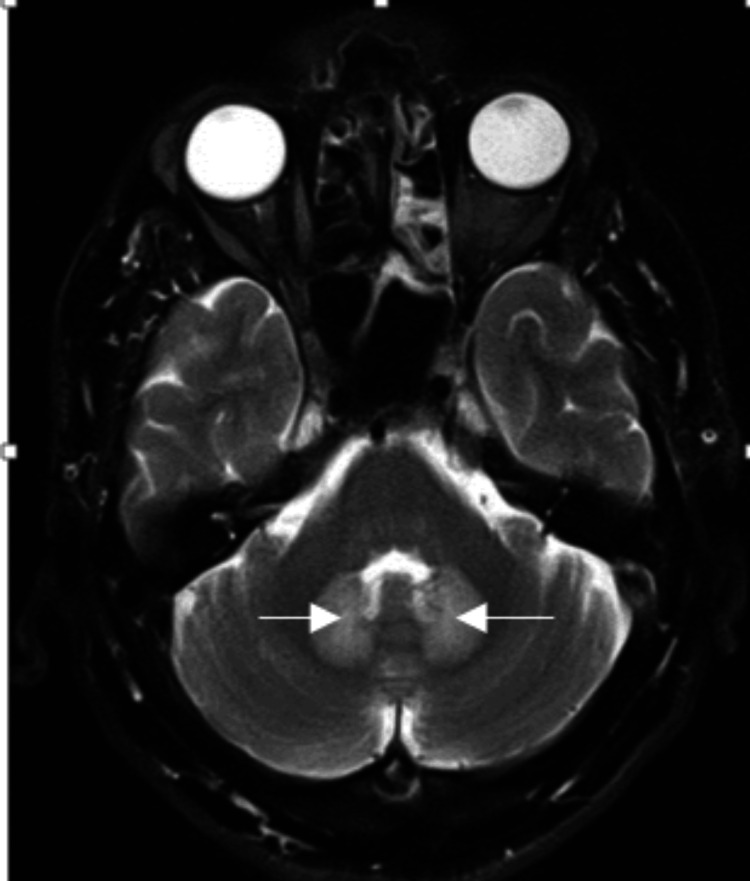
Axial T2 sequence showing bilateral dentate nuclei hyperintensity (white arrows).

Contrast-enhanced CT revealed well-defined, thick-walled collections with liquefactive necrosis in segments IVa, V, and VIII. A possibility of paraneoplastic syndrome secondary to hepatocellular carcinoma which is known to cause carcinomatous neuropathy was considered due to increasing abscess size. A diagnostic aspiration of the abscess revealed no evidence of cellular atypia, and tumor markers workup was within normal limits, showing serum alpha-fetoprotein levels of 8.1 ng/mL, cancer antigen 19.9 of 13.28 U/mL, and carcinoembryonic antigen of 1.05 ng/mL. While in the hospital, the patient developed bilateral cerebellar signs in the form of dysdiadochokinesia, past pointing, along with stance and gait ataxia. Subsequent MRI of the brain revealed bilateral symmetrical T2 fluid-attenuated inversion recovery hyperintensities in the dentate nucleus, suggestive of toxic or metabolic encephalopathy (Figure 4).

Metronidazole was discontinued and ciprofloxacin and supportive treatment with thiamine, methylcobalamin, and folic acid was started. A significant improvement with near-complete resolution of sensory symptoms and cerebellar signs was noted over the subsequent days. At follow-up after a month, repeat NCS revealed bilateral median nerve entrapment neuropathy at the wrist and sensory neuropathy of both lower limbs. Serial abdominal ultrasonography revealed gradually resolving abscess, with complete resolution achieved over eight months.

## Discussion

A sudden increase in the size of liver abscesses while on treatment would raise a suspicion of hepatocellular malignancy. The association between hepatocellular carcinoma and peripheral neuropathy, particularly in the context of paraneoplastic syndromes, is relatively rare [3]. The presence of anti-Hu antibodies is typically associated with small-cell lung carcinoma but has also been detected uncommonly among cases of hepatocellular carcinoma accompanied by paraneoplastic peripheral neuropathy [3]. Drugs used in the treatment of liver abscesses such as metronidazole are also known to cause peripheral neuropathy [4].

In instances of peripheral neuropathy occurring in the background of a sudden increase in the size of liver abscess, clinicians should conduct a thorough evaluation to determine the cause of neuropathy and ascertain whether it is due to paraneoplastic manifestation or due to other causes such as drugs, as noted in the present case. Neurotoxicity associated with metronidazole can manifest as isolated peripheral neuropathy, cerebellar toxicity, gait disturbances, weakness, confusion, dysarthria, ataxia, and dysmetria [5].

Although the exact cause of metronidazole-induced peripheral neuropathy remains unclear, several mechanisms have been proposed in this regard. Rao et al. suggested the free radicals produced during the metabolism of metronidazole as the likely cause of nerve damage [6]. Bradley et al. proposed that metronidazole and its metabolites can bind to RNA, leading to the inhibition of protein synthesis and subsequent axonal degeneration of nerve fibers [4]. Another proposed mechanism is the enzymatic conversion of metronidazole to a thiamine analog which might lead to a neuropathy resembling a nutrition deficiency-related condition [7].

Neurological complications have been reported to occur within a few days to weeks after treatment initiation. Kuriyama et al. [8] and Kim et al. [9] observed neurotoxicity symptoms within two hours and seven days of metronidazole administration, respectively. The longest duration for the onset of symptoms recorded was four months [10]. Our patient developed symptoms three weeks after the initiation of therapy, and cerebellar toxicity was noted in the fourth week. Management involves prompt discontinuation of metronidazole, which often leads to symptom resolution.

The use of metronidazole, in combination with ciprofloxacin or cephalosporin, is a widely practiced empirical therapy for liver abscess, a practice supported by existing clinical guidelines [11]. Several factors can be considered to cause the neuropathic manifestations in the present scenario. Although vitamin B complex deficiency could have been a contributing factor probably due to an acute depletion caused by antibiotic usage, this was unlikely in our case as the patient was already receiving B complex vitamin supplementation along with his diabetic medications even before the presentation. While diabetes mellitus is a common cause of neuropathy, the patient did not exhibit neuropathic symptoms at the time of initial presentation for the liver abscess. These symptoms developed only after the initiation of metronidazole treatment. The temporal correlation between metronidazole discontinuation and symptom improvement strongly suggests a possible link to the drug rather than solely to the vitamin supplements. Additionally, paraneoplastic causes were also excluded, reinforcing the likelihood of metronidazole-induced neuropathy. This supports our conclusion regarding the drug’s role in the development of neuropathy.

## Conclusions

This case highlights the importance of ruling out malignancy and drugs as a cause of neurological manifestations in a patient with a sudden increase in the size of a hepatic abscess despite treatment. The strong consideration of metronidazole-related neurological manifestations involving cerebellar manifestations or neuropathic manifestations, especially in prolonged duration of treatment, is important.
